# The Pleasantness of Visual Symmetry: Always, Never or Sometimes

**DOI:** 10.1371/journal.pone.0092685

**Published:** 2014-03-21

**Authors:** Anna Pecchinenda, Marco Bertamini, Alexis David James Makin, Nicole Ruta

**Affiliations:** 1 Sapienza University, Department of Psychology, Sapienza University, Rome, Italy; 2 Department of Experimental Psychology, Eleanor Rathbone Building, University of Liverpool, Liverpool, United Kingdom; University of Gent, Belgium

## Abstract

There is evidence of a preference for visual symmetry. This is true from mate selection in the animal world to the aesthetic appreciation of works of art. It has been proposed that this preference is due to processing fluency, which engenders positive affect. But is visual symmetry pleasant? Evidence is mixed as explicit preferences show that this is the case. In contrast, implicit measures show that visual symmetry does not spontaneously engender positive affect but it depends on participants intentionally assessing visual regularities. In four experiments using variants of the affective priming paradigm, we investigated when visual symmetry engenders positive affect. Findings showed that, when no Stroop-like effects or post-lexical mechanisms enter into play, visual symmetry spontaneously elicits positive affect and results in affective congruence effects.

## Introduction

There is evidence that people prefer symmetry. This is true not only for aesthetic appreciation of works of art [1], [2] but also for perceived attractiveness: observers find symmetrical faces and bodies more attractive [3], [4].

To explain the role of symmetry in attractiveness it has been argued that, in the natural world, bilateral symmetry is an indicator of gene quality [5], [6]. This view has been criticised [7] and some evidence of publication bias has been reported [8]. Another account attributes the preference for visual symmetry to the fact that our visual system processes symmetry efficiently. Indeed, there is much evidence for the fast and efficient processing of symmetry [9], [ for reviews see 10], [ 11]. Most salient are bilateral symmetry patterns that have a vertical axis [9], [ 11]–[14], [ but see 15] and are presented within the context of a closed region [16]–[18].

If the visual system is tuned to bilateral symmetry, perhaps because this regularity has a special role in perception of shape [19], then preference for symmetry may be a by-product of efficient processing. Specifically, the fluency hypothesis says that individuals' preferences for symmetry are due to the positive affect engendered by processing fluency [7], [20]–[22]. Indeed, Reber, Schwarz, and Winkielman [23] argue that what is special about symmetric objects is that they contain less information and are easier to process.

There is much evidence that fluently processed objects (i.e., faster and more accurately) are preferred to objects that are processed less fluently, [24]–[27]. However, previous work had typically involved explicit ratings and we know that explicit measures might be weakened by factors such as desirability and/or accessibility. Furthermore, explicit ratings do not establish whether the preferences formed spontaneously and incidentally, that is, without the need to focus on the aesthetic merit [28]. In contrast, recent studies have used implicit measures to overcome some of these limitations.

### Implicit preferences for visual symmetry

Winkielman and Cacioppo [26] provided evidence that processing fluency results in hedonic experience as indexed by indirect measures of affect, like increased electromyographic activation of the *Zygomatic Major* muscle involved in smiling. These findings were recently replicated in a study in which participants categorized patterns with two types of regularities (reflection and rotation symmetry) and random patterns while measuring EMG activity at the *Zygomatic Major* muscle and ERP components [29]. Findings showed greater activity of the *Zygomatic Major* muscle and lower amplitude of the Sustained Posterior Negativity (SPN) – a symmetry-related ERP component – when participants categorized symmetric patterns based on regularity. Interestingly, *Zygomatic Major* muscle activity reversed when participants treated the random patterns as targets of their categorizations. Thus, greater *Zygomatic Major* muscle activity for reflection patterns is consistent with the idea that symmetry is experienced as positive. However, that changing instructions could reverse this effect casts doubts on whether it occurs *spontaneously* or it depends on symmetry being the focus of the classification task, i.e., the target category.

Further evidence showing that when symmetry is the focus of the classification task it becomes associated with positive affect comes from studies using the Implicit Association Test (IAT), [30], [31]. Makin, Pecchinenda, and Bertamini [32] presented dot-patterns which were either symmetrical or random, and words which were either positive or negative. On compatible blocks, one key was used for symmetry or positive words, and the other key was used for random or negative words. On incompatible blocks, the response mapping was reversed (symmetry and negative on one key, random and positive on the other key). The difference in response time (compatible blocks versus incompatible blocks) is an index of the strength of association between positive words and reflection symmetry. Participants were faster when the same key was used to classify reflection dot-patterns and positive words and another key was used for random dot-patterns and negative words compared to when the reverse mapping was used. This pattern of IAT results indicates a preference for reflection symmetry dot-patterns over random patterns. This was replicated with other regularity types [33], with associations between symmetry and positive, high arousal words [34], as well as with a multidimensional IAT [35]. However, the evidence of a link between symmetry and positive, high arousal affect challenges the account that symmetry is liked because it is processed fluently (i.e., the fluency hypothesis). In fact, non fluent processing is more arousing than fluent processing.

In a recent study, Bertamini, Makin, and Pecchinenda [36] have used the affective priming paradigm to investigate hedonic responses to symmetry. Symmetric or random patterns served as primes and positive or negative words served as targets in a word classification task. In this paradigm participants' attention is focussed on target stimuli and primes are presented incidentally: The implicit measure is given by the processing advantage due to affective congruence between prime and target stimuli. Affective congruence effects, in terms of interference from random patterns on positive words (rather than of facilitation from symmetric patterns) were observed. More importantly, these effects emerged only when the visual regularity of the primes (bilateral reflection symmetry but also rotational symmetry) had to be actively classified. These results once again suggest that activation of the categories “symmetry” and “random”, rather than visual processing of symmetry in a pattern, is key to an affective response.

In summary, evidence shows that when indirect measures of affect are used, visual symmetry is linked to positive affect, provided the regularity of the patterns is the focus of the classification task.

### Neural correlates of visual symmetry

It is interesting to note that research on the neural correlates of symmetry processing shows that visual symmetry modulates neural activity regardless of attention. Jacobsen and Hoöfel [37] recorded ERPs while participants viewed and categorized symmetric and random visual patterns. They observed the Sustained Posterior Negativity (SPN) that was more negative for symmetric than for random patterns. Interestingly, this finding was replicated in a study in which explicit categorization of the visual stimuli was not required [28]. Makin, Rampone, Pecchinenda, and Bertamini [38] found that the SPN was comparable when participants performed a categorization task or an oddball task, which does not require to focus on symmetry. In all conditions there was greater SPN amplitude for visual regularities (reflection, rotation, and translation symmetry) compared to random patterns.

That processing visual regularities is associated to specific neural markers, independently of whether the task requires participants to focus on symmetry, is at odds with evidence linking visual symmetry to positive affect. This evidence shows that only when participants categorize stimuli based on regularity, visual symmetry is associated to positive affect.

### The present research

We conducted 4 experiments using variants of the affective priming paradigm [39] to test which conditions give rise to congruence effects due to the positive affect elicited by visual symmetry. The affective priming paradigm has multiple advantages: It allows us to use symmetric or random patterns as primes presented incidentally, we can manipulate participants' attention by asking them to perform a task on the prime stimuli, and it allows us to use different stimuli as targets to rule out factors other than positive affect.

Experiment 1 used the typical affective priming paradigm with an evaluative task and responses via a key press. Experiment 2 introduced a delayed categorization on the dot-patterns used as primes to assess whether affective congruence effects depends on participants' attention being explicitly focused on the symmetry of the dot-patterns.

In the affective priming paradigm with responses to targets by key presses as in Experiments 1 and 2, facilitation or interference effects could be engendered either by the congruence of affect elicited by prime and target but also by the congruence of responses elicited by prime and target stimuli. In Experiment 3, participants read the target-words aloud. We measured voice onset latency. This methodology is similar to that used in previous studies with words [40], [41] as well as with nonrepresentational sounds and shapes [42]. The advantage of this task is that as response to each target is unique (i.e., reading the target-word), Stroop-like mechanism linked to response facilitation or interference cannot play a role in engendering congruence effects between prime and target. Therefore, if visual symmetry activates positive affect, it will result in processing facilitation of affectively related information [43]–[45]. Finally, Experiment 4 used the Affect Misattribution Procedure (AMP), [46] which is another variant of the affective priming paradigm. In this procedure, symmetric and random patterns are presented incidentally as primes but the targets are unfamiliar and neutral.

## Experiment 1

**Figure 1:**
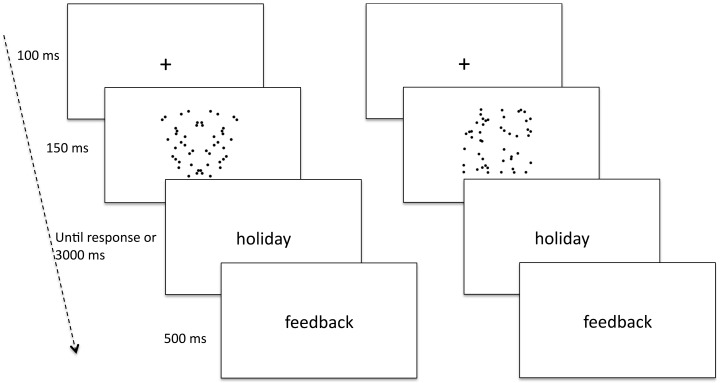
Examples of stimuli and sequence of events used in the affective priming procedure. An example of congruent trial with a symmetric pattern as prime and a positive target-word is shown in the left panel. An example of incongruent trial with a random pattern as prime and a positive target-word is shown in the right panel.

We used the affective priming paradigm with patterns of random dots or with patterns of dots reflected around a vertical axis as primes, followed by positive and negative words as targets (see Figure 1). If symmetry engenders positive affect, congruence effects should be observed when positive target-words are paired with reflection dot-patterns. This should be reflected in shorter RTs to words preceded by congruent, symmetric patterns compared to when preceded by incongruent, random patterns.

This methodology is similar to that adopted in Bertamini et al., [36] with two differences. We did not use high-contrast black and white patterns formed by a small number of elements. These stimuli were originally designed for EEG studies and the random patterns may have contained a high degree of regularity. The current study instead, used sparse dot patterns, as illustrated in Figure 1. In addition, we counterbalanced prime-target assignment in two versions of the task so that an identical symmetric prime paired with a positive word in one version of the task was paired with a negative word in the other version of the task. This allowed us to assess whether past findings were specific to the stimuli and methodology used previously.

### Methods

#### Ethics Statement

All experiments had received approval by the ethics committee of the Department of Psychology, Faculty of Medicine and Psychology, Sapienza University.

### Participants

Twenty participants (age *M* = 26 years, range 22 to 31, 10 males, 10 females) volunteered and gave written informed consent before taking part in the experiment. They had normal or corrected to normal vision and were naïve with respect to the experimental hypotheses.

#### Apparatus

Participants sat in a quiet room in front of a 17″ CRT monitor (1024×768 pixels, 60 Hz) connected to a Dell Precision T15000 (One Intel Core i7-870; 2.8 GHz, 8 MB, 95 W, QC). Stimuli were presented using E-Prime Version 2.0 Professional software [47], which also recorded participants' responses. Prime-stimuli were comprised of 144 black dots on a white background, half of which were symmetric and had a reflection around the vertical axis. The other half were random stimuli and were not constrained except that there were an equal number of dots in each side of the vertical axis. Therefore, the type of pattern used as prime (reflection or random) was specified by the relationship between the two halves, and it would be impossible to distinguish between conditions from just one side.

Targets were 144 words selected from an Italian translation and validation of the Affective Norms for English Words (ANEW), [48]. For the validation, the translated words were judged by an independent group of 100 students in terms of valence and arousal on a 9-point scale [49]. There were 72 positive words (Valence *M* = 7.00; *SE* = .55; Arousal *M* = 4.70; *SE* = 1.06) and 72 negative words (Valence *M* = 2.33; *SE* = .43; Arousal *M* = 5.40; *SE* = 1.10). The selected words differed on valence ratings, *t*(71) = 271.6, *p*<.001, but were matched as much as possible on word length and on arousal ratings.

### Procedure

After each participant had given informed written consent, they sat in front of a computer in a dimly lit room and completed 24 practice trials followed by 144 trials divided in two blocks of 72 trials each. There were 36 symmetric dot-patterns followed by positive words and 36 symmetric dot-patterns followed by negative words. Similarly, for the random dot-patterns, 36 were followed by positive words and 36 were followed by negative words. Two different versions of the task were used to counterbalance between-subjects prime-target assignment, so that positive words presented after symmetric dot-patterns in one version of the task were presented after random dot-patterns in the other version of the task.

Stimuli were presented in a new random order to each participant. A trial had the following sequence of events (see Figure 1): After a fixation mark (+) presented for 100 ms, a dot-pattern was presented for 150 ms, followed by a target-word, which remained on screen until response or 3000 ms had elapsed. A response-feedback (correct, incorrect, no response detected) followed for 500 ms. The inter trial interval (ITI) was variable and ranged from 500 to 1500 ms. Participants responded to target-words using a USB keyboard and by pressing the “U” and “B” keys (labelled “POS” and “NEG”) with the index and middle finger of the right hand. These keys were chosen because perpendicular to the left-right regularity of the dot-patterns. Key assignment to “positive” and “negative” was counterbalanced between-subjects in two versions of the task.

At the end of the affective priming task, participants completed a categorization task on the dot-patterns. They saw the 72 reflection and 72 random dot-patterns used as primes and classified them as symmetric or random by pressing the keys “D” or “S” labelled as “SIM” and “RAN”. Keys assignment was counterbalanced between participants.

#### Experimental Design

A 2 (Prime: Symmetric *vs.* Random patterns) ×2 (Target: Positive *vs.* Negative words) within-subject design was used.

### Data Analyses and Results

**Figure 2:**
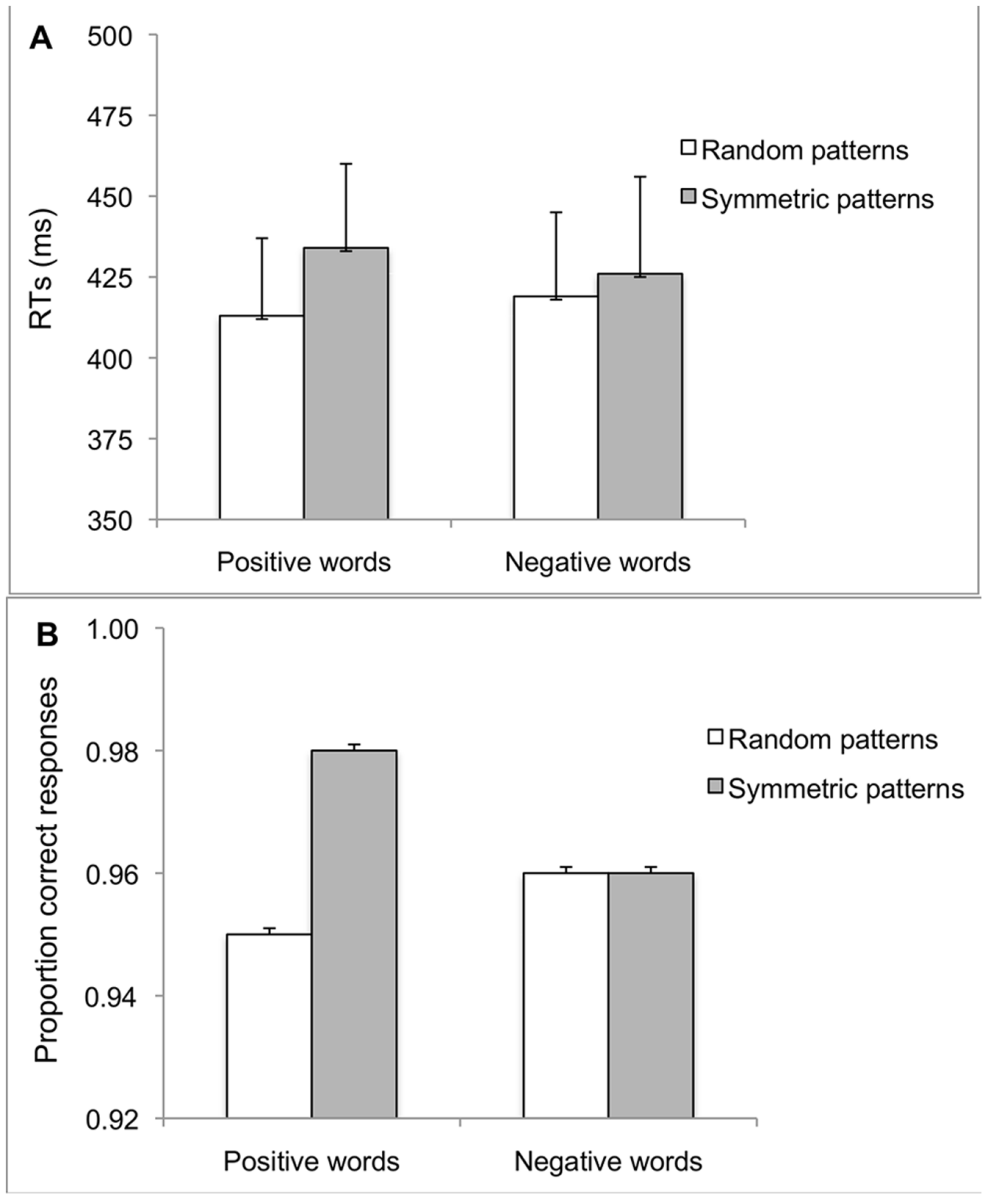
Experiment 1: Mean RTs (2a) and proportion of correct responses (2b) to positive and negative word-targets preceded by symmetric and random dot-primes in the typical affective priming paradigm with responses by key-presses. Error bars = ±1 S.E.M.

After removing RTs shorter than 120 ms or longer than 1200 ms (1%), the median RTs were calculated for each experimental condition on correct responses. RTs to target-words were analysed using a 2×2 ANOVA for repeated measures. Results for RTs showed that neither the main effect of Prime, *F*(1, 19) = 2.80, *p* = .110, the main effect of Target, *F*(1, 19) = .017, *p* = .897 nor the interaction, *F*(1, 19) = 1.014, *p* = .327 were significant (see Figure 2a).

ANOVA results for proportion of correct responses showed that neither the main effect of Prime, *F*(1, 19) = 1.977, *p* = .176 nor the main effect of Target, *F*(1, 19) = .219, *p* = .645 were significant. The interaction was significant, *F*(1, 19) = 6.342, *p* = .021, partial η^2^ = .25 (see Figure 2b). This was due to greater response accuracy when Positive words were preceded by Symmetric dot-patterns, (*M* = .98; *SE* = .007) compared to when preceded by Random dot-patterns, (*M* = .95; *SE* = .010), *t*(19) = 3.00, *p* = .007. Response accuracy for Positive words preceded by Symmetric dot patterns tended to be greater than response accuracy to Negative words preceded by Symmetric dot-patterns, (*M* = .96; *SE* = .012), *t*(19) = 2.04, *p* = .055.

Data of the categorization task performed on the dot-patterns at the end of the affective priming task served as the manipulation check that symmetric dot-patterns were indeed categorized more fluently than random dot-patterns. A t-test revealed that participants were faster in categorizing the dot-patterns as symmetric, (*M* = 410, *SE* = 26) than in categorizing the dot-patterns as random, (*M* = 438, *SE* = 30), *t*(19) = 2.23, *p* = .032. There was no difference in the accuracy of categorization for symmetric, (*M* = .94, *SE* = .016) and random, (*M* = .93, *SE* = .015) dot-patterns, *t*(19) = .625, *p* = .539.

### Discussion

In Experiment 1 we used the affective priming paradigm with key-presses to an evaluative task, and manipulated the pairing of symmetric and random dot-patterns with positive and negative words. Under these conditions, if symmetry is experienced as positive, responses to positive target-words should be faster when preceded by symmetric dot-patterns. Yet, as in Bertamini et al. [39] no affective congruence effects were observed on response times, despite the fact that the categorization task showed the typical pattern of faster responses to symmetric dot-patterns. In contrast, we observed affective congruence effects on response accuracy. Participants were more accurate in responding to positive target words when they were preceded by symmetric dot-patterns. Therefore, although symmetric dot-patterns are processed more fluently when explicitly categorized, this fluency of processing does not result in affective congruence effects on response times but results in greater response accuracy to positive target-words.

These results are difficult to interpret. Moreover, the significant congruence effects for accuracy could be due to Stroop-like interactions between the responses elicited by the two categories of stimuli. In addition, the null result on response times may be because visual symmetry does not elicit positive affect or because visual symmetry is not a salient characteristic of the stimuli we used. Indeed, one may argue that participants did not see the symmetry at all, because there is some evidence that symmetry does not pop out in visual search tasks [11], [50], [51].

## Experiment 2

In this study we investigated whether affective congruence effects emerge when participants explicitly focus their attention on the regularity of the dot-patterns, leaving aside for the moment the issue of whether or not Stroop-like mechanisms play a role. After classifying the target-words as positive or negative, participants performed a delayed categorization on the primes based on whether they were symmetric or random.

### Participants

Twenty participants (age *M* = 24 years, range 19 to 25, 5 males, 15 females) who had not taken part in the previous experiment, volunteered after giving informed written consent. They had normal or corrected to normal vision and were naïve with respect to the experimental hypotheses.

### Procedure

The experimental procedure and stimuli were as in Experiment 1, with one exception relative to the task's instructions: Participants were instructed to assess whether the dot-patterns used as primes were symmetric or random. They were informed that after they responded to the target-word as being positive or negative, a question mark would appear on the computer screen, which prompted them to press one of two keys on the keyboard with their left hand if the dot-pattern they had just seen was symmetric (“D”) and another key (“S”) if it was random. The keys were labelled “SIM” and “RAN”. Keys assignment was counterbalanced between participants.

### Data Analyses and Results

**Figure 3:**
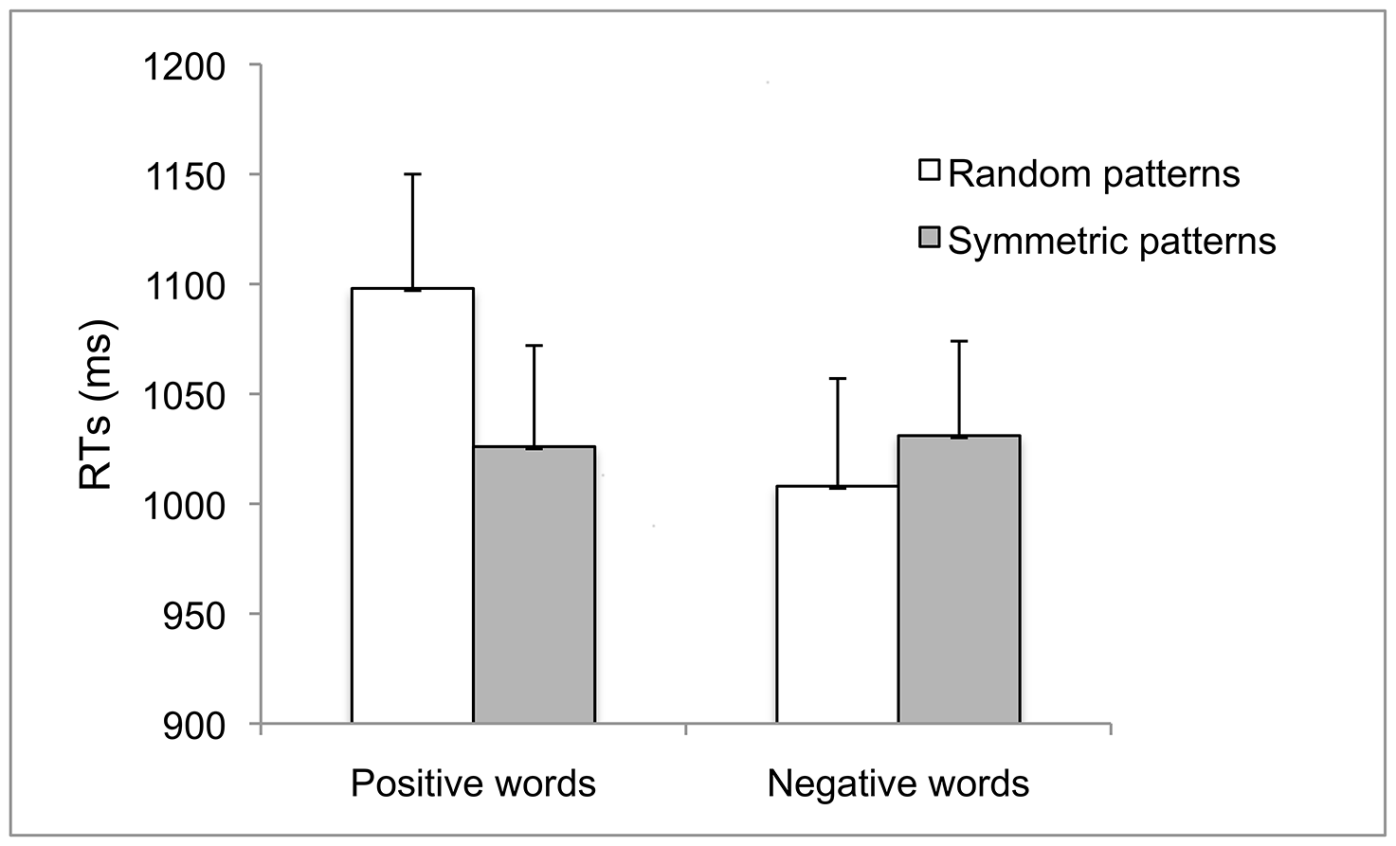
Experiment 2: Mean RTs to positive and negative word-targets preceded by symmetric and random dot-primes in the affective priming paradigm with delayed categorization on primes. Error bars = ±1 S.E.M.

As participants performed two tasks combined, RTs in this experiment were much longer than those of Experiment 1. After having removed RTs shorter than 120 ms or longer than 1500 ms (1.1%) the median RTs for each experimental condition were computed on correct responses. Data were analysed using a 2×2 ANOVA for repeated measures. Results showed that neither the main effect of Prime, *F*(1, 19) = 1.64, *p* = .215 nor the main effect of Target, *F*(1, 19) = 3.16, *p* = .092 were significant. However, the interaction was significant, *F*(1, 19) = 11.90, *p* = .003 partial η^2^ = .385, (see Figure 3). This was due to slower RTs for Positive words when preceded by Random dot-patterns, (*M* = 1098; *SE* = 52) than when preceded by Symmetric dot-patterns, (*M* = 1026; *SE* = 49), *t*(19) = 3.17, *p* = .005. In contrast, RTs for Negative words preceded by Random dot-patterns, (*M* = 1008; *SE* = 46) did not differ from when preceded by Symmetric dot-patterns, (*M* = 1032; *SE* = 44), *t*(19) = 1.01, *p* = .325. Results on proportion of correct responses showed no significant main effects or interaction.

For the delayed symmetry categorization performed on primes after participants had responded to the targets, t-test results revealed that participants were still faster in evaluating the dot-patterns as symmetric, (*M* = 420; *SE* = 37) than in evaluating them as random, (*M* = 446; *SE* = 44), *t*(19) = 1.75, *p* = .04 (one tail). There was no difference in the accuracy of categorization for symmetric, (*M* = .85, *SE* = .036) and random dot-patterns, (*M* = .88, *SE* = .028), *t*(19) = 1.12, *p* = .257.

### Discussion

In Experiment 2 we used the affective priming paradigm and introduced a delayed categorization task on the prime to focus participants' attention on symmetry. Under these conditions, affective congruence effects were observed. These effects were similar to those reported by Bertamini et al. [36]: longer response times to positive targets when preceded by random primes. It is unlikely that the mechanism underlying this effect is the positive affect engendered by symmetric primes. That is, even when participants' attention was focused on stimulus regularity, no affective congruence effects – namely facilitation with shorter responses – to positive words when preceded by symmetric primes, were observed.

These results allow to rule out that symmetry needs to be under the focus of attention to engender positive affect. Instead, they hint at the possibility that the dot-patterns used as primes may activate something else besides affect. In fact, in the affective priming paradigm with responses to targets by key presses and an evaluative task, congruence effects can be observed when primes and targets share a response based on their valence or on some other category, or even if the participants recoded the task as requiring a yes/no response. These congruence effects could be due to facilitation of the congruent response, to interference by the incongruent responses, or both [52]. In these cases however, affective congruence effects are best accounted by Stroop-like effects and post-lexical mechanisms [53]. One way of ruling out Stroop-like mechanisms consists in avoiding one-to-one mapping between target responses and valence whereas judgmental tendencies do not play a role when responses are not clearly affirmative or non-affirmative. This was addressed in Experiment 3.

## Experiment 3

In Experiment 3 we used the affective priming paradigm with vocal responses to target-words. In this task, the response depends on the unique identity of the stimulus, which prevents assigning prime and target of the same valence to the same response. Therefore, the presentation of a positive prime cannot pre-activate the correct response as the response based on the identity of the target (i.e., read the word aloud) and that based on the prime are always different [41].

Participants completed an affective priming task as in Experiment 1 with the only difference that this time, they responded to the target words by reading them out into a microphone.

### Participants

Sixteen participants (age *M* = 23 years, range 19 to 32, 4 males, 12 females), who had not participated in the previous experiments, volunteered and gave informed written consent before taking part in the experiment. They had normal or corrected to normal vision and were naïve with respect to the experimental hypotheses.

### Procedure

The experimental procedure and stimuli were as in Experiment 1, with the exception that participants were instructed to read the target-word aloud into a microphone and no response feedback was provided. Vocal RTs were recorded using a microphone connected to E-Prime Serial Response Box with voice activation key with trip level and volume adjusted individually for each participant at the beginning of the experiment.

At the end of the affective priming task, participants completed a categorization task on the dot-patterns. The procedure was as in Experiment 1.

### Data Analyses and Results

**Figure 4:**
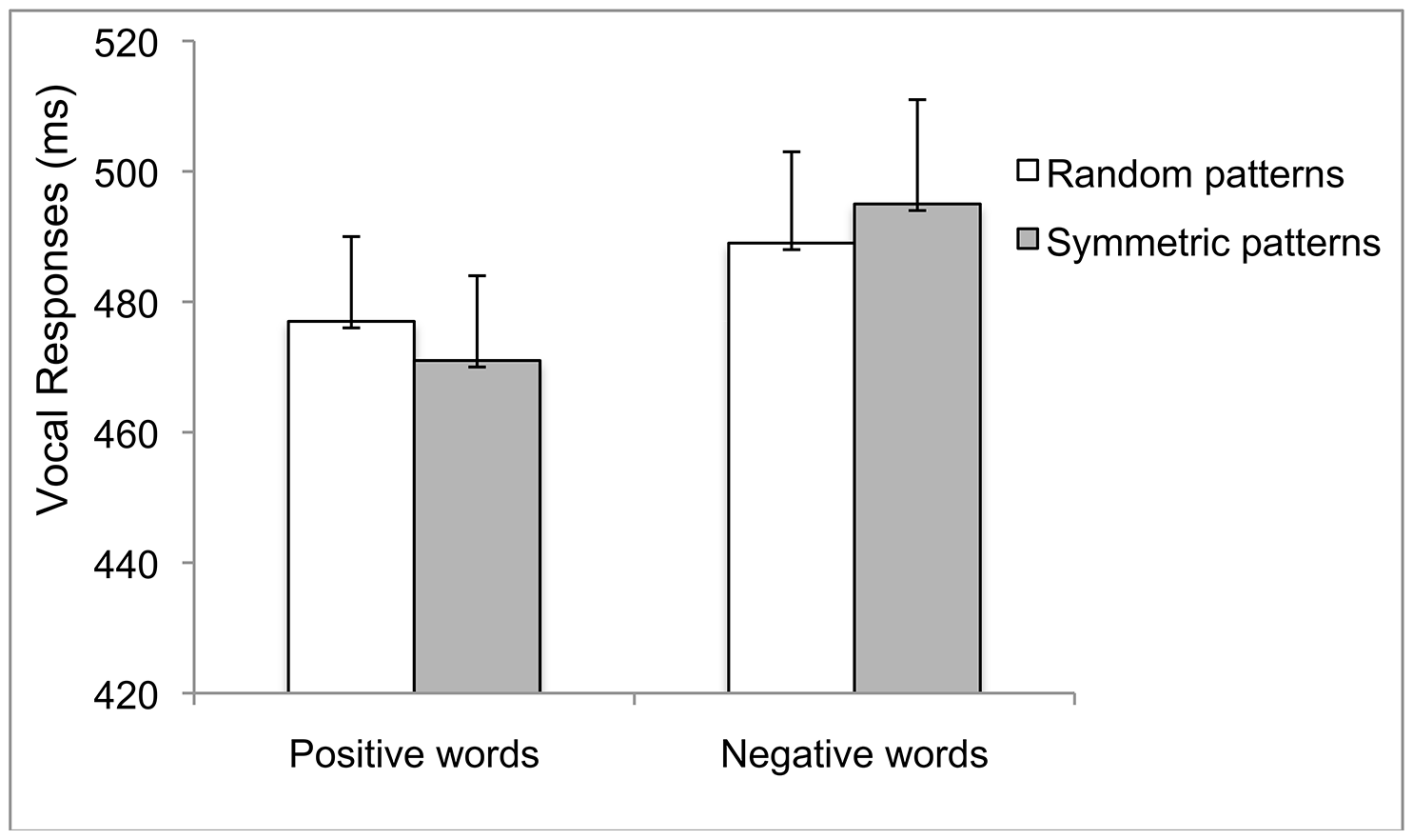
Experiment 3: Mean vocal RTs to positive and negative word-targets preceded by symmetric and random dot-primes in the affective priming paradigm with a pronunciation task. Error bars = ±1 S.E.M.

After having removed values shorter than 120 ms or longer than 1200 ms (less than 1%), median vocal RTs for each experimental condition were computed. Data were analysed using a 2×2 ANOVA for repeated measures. Results showed no main effect of Prime, *F*(1, 15) = .002, *p* = .963. The main effect of Target was significant, *F*(1, 15) = 22.30, *p*<.000 partial η^2^ = .598, indicating faster vocal RTs to Positive Words, (*M* = 474, *SE* = 13) than to Negative Words, (*M* = 492, *SE* = 15). This main effect was qualified by an almost significant interaction, *F*(1, 15) = 4.41, *p* = .053, partial η^2^ = .227 (see Figure 4). t-test results showed that vocal RTs to Positive Words preceded by Symmetric dot-patterns were faster, (*M* = 471; *SE* = 13) than vocal RTs to Negative Words preceded by Symmetric dot-patterns, (*M* = 495; *SE* = 16), *t*(15) = 4.71 *p*<.000. Similarly, vocal RTs to Positive Words preceded by Random dot-patterns were faster, (*M* = 477; *SE* = 13) than vocal RTs to Negative Words preceded by Random dot-patterns, (*M* = 489; *SE* = 14), *t*(15) = 2.46 *p* = .026. However, whereas this pattern resulted in a significant difference between vocal RTs to Positive Words when preceded by Symmetric dot-patterns than when they were preceded by Random dot-patterns, *t*(15) = 2.34 *p* = .003, the same comparison for Negative Words preceded by Symmetric dot-patterns or by Random dot-patterns did not reach statistical significance, *t*(15) = 1.10, *p* = .289.

For the categorization task performed on the dot-patterns at the end of the affective priming task, t-test results revealed that participants were faster in categorizing the dot-patterns as Symmetric, (*M* = 628, *SE* = 32) than in categorizing the dot-patterns as Random, (*M* = 663, *SE* = 34), *t*(15) = 2.43, *p* = .028. There was no difference in the accuracy of categorization for symmetric, (*M* = .97, *SE* = .005) and random dot-patterns, (*M* = .95, *SE* = .013), *t* (15) = 1.29, *p* = .214.

### Discussion

The results of Experiment 3 showed that when using the affective priming paradigm with vocal responses to targets, affective congruence effects were observed. There were faster vocal responses to positive target-words preceded by symmetric dot-patterns. According to current perspectives in aesthetics, this positive affect is a by-product of the fluency experienced when perceiving symmetry. Indeed, our dot-patterns showing reflection symmetry were easily categorized as symmetric with much faster response times than those for dot-patterns not showing this regularity. Therefore, when using a task that allowed for incidental presentation of patterns with or without visual symmetry (but at the same time did not allow for Stroop-like effects to play a role) there was evidence of affective congruence between positive targets and symmetric primes. This effect was due to symmetric primes engendering positive affect and occurred spontaneously, without explicitly directing participants' attention to the regularity of the primes.

It should be noted that in the present experiment responses to positive targets were overall faster than responses to negative targets. While this is a well known finding in literature [54], [55] one may argue that as both symmetric patterns and positive words are processed more fluently, if people implicitly associated fluent categories of stimuli, this matching mechanism could give rise to congruence effects [56]. Although this reasoning implies that the same matching mechanism could apply to the less fluent categories of stimuli, as an ultimate test of the hypothesis that visual symmetry elicits positive affect, we used yet another variant of the affective priming paradigm, this time with unfamiliar and affectively neutral targets [57]. In Experiment 4 we used the Affect Misattribution Procedure (AMP), [46] which is a reliable tool to assess individuals' attitudes.

## Experiment 4

In the AMP, prime stimuli assumed to have positive or negative valence are presented for a brief period of time followed by an unfamiliar target that is affectively neutral. Participants' task is to evaluate the target as visually pleasant or visually unpleasant by pressing one of two keys.

The rationale of the AMP is that when participants are asked to make pleasantness judgments of otherwise neutral target-stimuli, if they cannot use any other information to make their judgments, they will use the affective information elicited by the prime and misattribute it to the targets. This misattribution occurs in spite of the instructions to try to avoid any possible influence of the prime on their judgments of the target [46], [58].

### Participants

Eighteen participants (age *M* = 23 years; range 19 to 40, 9 males, 9 females) volunteered and gave informed written consent before taking part in the experiment. They had normal or corrected to normal vision and were naïve with respect to the experimental hypotheses.

#### Apparatus

The experimental set-up was as in Experiment 1, with the following exceptions: Primes were 72 different dot-patterns (36 symmetric and 36 random) whereas targets were 72 different Chinese pictograms from Payne et al. [46].

### Procedure

**Figure 5:**
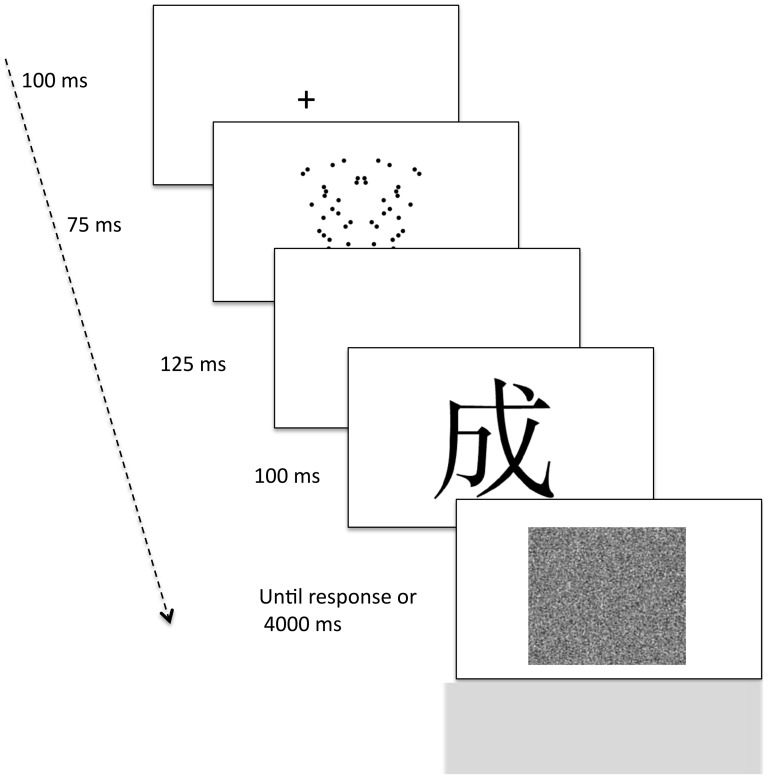
Experiment 4: Examples of stimuli and the sequence of events used in the Affect Misattribution Procedure.

After 18 practice trials, participants completed 72 trials divided in two blocks of 36 trials each. Stimulus presentation followed a different random order for each participant. A typical trial started with a fixation mark (+) for 100 ms, followed by a dot-pattern for 75 ms, followed by a blank for 125 ms, and a Chinese pictogram for 100 ms. After the pictogram, a mask consisting of black and white “noise” appeared and remained on screen until the participant responded or 4000 ms had elapsed (see Figure 5). A feedback “no response was detected” was given only if participants did not respond within the allowed time and remained on screen for 500 ms. The ITI was 1000 ms.

Two different versions of the task were created to counterbalance between-subjects prime-target assignment so that if a Chinese pictogram was assigned to a random dot-pattern in one version of the task, it was assigned to a symmetric dot-pattern in the other version of the task. As in Payne et al. [46] participants were instructed that a dot pattern would first appear briefly, and represented the warning signal for the Chinese pictogram. Participants had to press a key if they found the Chinese pictogram to be visually pleasant, and another key if they found the Chinese pictogram to be visually unpleasant. They were warned that sometime having just seen a stimulus could influence their judgments and were instructed to try to avoid any influence of the dot-pattern on their judgments of the pictograms.

Participants responded by pressing the “U” and “B” keys on the keyboard, which were labelled as “pleasant” or “unpleasant”. Key assignment was counterbalanced between subjects.

### Data Analyses and Results

The proportion of Chinese pictograms judged as pleasant following each type of dot-patterns was computed. t-test results showed that participants judged as more visually pleasant Chinese pictograms preceded by a symmetric dot-pattern, (*M* = .60, *SE* = .03) compared to pictograms preceded by a random dot-pattern, (*M* = .50, *SE* = .03), *t*(17) = 2.14, *p* = .047. Therefore, Experiment 4 confirmed the presence of misattribution of positive affect generated by the visual symmetry of dot patterns.

## General Discussion

We conducted four experiments to compare affective responses to symmetric and random patterns. Previous studies had provided a complex picture showing that incidental presentation of visual symmetry does not spontaneously engender positive affect, as indexed by changes in EMG activity over the *Zygomatic Major* muscle, by response associations, and by affective congruence effects. However, when people classify symmetry, affective congruence effects can be found [36], [29]. Interestingly, the absence of spontaneous affective responses is unlikely to reflect the lack of visual processing altogether: ERPs produced by symmetry can be recorded regardless of whether participants' attention is focussed on this characteristic of the stimuli [38]. However, if neural correlates show that visual symmetry is detected spontaneously, why it would require attention to engender positive affect?

The present research shows that results critically depended on the underlying mechanism engendering affective congruence effects. In Experiment 1 we used the traditional affective priming paradigm with an evaluative task and responses by key-presses to positive and negative target-words. We found no affective congruence effects based on reaction time, although there was a change in error rate: symmetric patterns resulted in more accurate classification of positive words. Whereas the result on response accuracy together with the null result from the analysis of reaction time is in line with previous findings (experiments 2, 5, and 6) [36], they are difficult to interpret. This is because in the affective priming paradigm different mechanisms may underlie facilitation and interference effects, sometimes resulting in competing influences on behavioural measures. In addition, there is some evidence that symmetry elicits positive affect only when participants explicitly focus their attention on this feature of the stimuli [28], [36], [50], [59]. Therefore, in three further experiments we assessed whether participants' attention on symmetry is necessary for affective congruence effects to occur and the role of different mechanisms underlying congruence effects in the affective priming paradigm.

In Experiment 2 we explicitly brought participants' attention on symmetry by asking them to make a delayed categorization on the dot-patterns. Under these conditions, congruence effects were observed but they were due to interference effects with longer RTs to positive words preceded by random dot-patterns rather than to facilitation effects with shorter RTs to positive words preceded by symmetric primes. As interference effects are typically due to Stroop-like mechanisms, Experiments 3 and 4 used variants of the affective priming paradigm that did not allow for a match between the response activated by the prime and that activated by the target.

In Experiment 3 we used vocal response. Stroop-like effects do not play a role here because prime and target of the same valence are not assigned to the same response [41], [60]. Under these conditions, affective priming effects were observed for positive words preceded by symmetric dot-patterns. These congruence effects were due to the positive valence engendered by the primes, which facilitated processing of the target stimuli. In addition, as in Experiment 3 there was no requirement to evaluate the dot-patterns or the words, and participants' attention was not brought on to the relevance of symmetry for the task at hand, these results support the conclusion that participants processed symmetry spontaneously. Although a note of caution is necessary due to the small sample size of Experiment 3, this is not a severe problem considering that we used a within-subject design, our participants were not pre-selected but represented a random sample of the students population and vocal responses at this task are characterized by less noise and inter-individual variability.

Finally, Experiment 4 provided further evidence that symmetric patterns elicit positive valence by using the Affect Misattribution Procedure with unfamiliar neutral targets. In this case, the positive affect elicited by the brief presentation (75 ms) of the primes is (mis)attributed to the unfamiliar neutral targets. Recently, the debate on the use of the AMP has been centred on whether the effects are solely affective or they can also be engendered by semantic misattribution [61], and whether individuals are aware of their affect misattribution [62], but see [63]. It should be noted that, for our purposes, this distinction is not critical. Firstly, the primes as well as the targets we used were relatively abstract and unfamiliar to our participants who did not know Chinese. Secondly, our research question was not whether individuals are or not aware that their preferences for some pictograms are due to the misattribution of affect engendered by primes. Rather, we were interested in whether visual symmetry spontaneously elicits positive affect or whether it requires that participants explicitly focus on this characteristic of the visual patterns. The findings of Experiment 4 indicate that symmetric dot-patterns *spontaneously* elicit positive affect, even when this is assessed using unfamiliar neutral targets and prime and target stimuli cannot be matched on fluency [56].

Nevertheless, one may argue that fluency of processing is only one of the possible factors that underlie our preferences for symmetry. Factors such as the insight following detection of a gestalt-like pattern (i.e., the Aesthetic Aha-Effect) [64] may modulate this relationship. Notwithstanding this, our findings contribute to the fluency literature and to the literature on the role of visual symmetry in aesthetics [23] in several important ways. Firstly, by showing that congruence effects observed with vocal responses in the affective priming paradigm or with affect misattribution to unfamiliar pictograms in the AMP reliably measure affect that arises *spontaneously* from differences in perceptual fluency by visual symmetry. This is consistent with a recent account in psychology of aesthetics, suggesting that people are sensitive to the efficiency or fluency of their own cognitive operations [21], and that people have a preference for stimuli that are fluently processed [25], [65]. Secondly, the present work clarifies why implicit measures used to assess the positive affect elicited by visual symmetry may at times provide mixed findings depending on the underlying mechanisms called upon by the tasks used. In conclusion, the present findings clarify some important issues about aesthetic processing by showing that visual symmetry in novel, abstract patterns *spontaneously* engenders positive affect, which can be measured implicitly and does not rely on attention being focused on symmetry.
